# Hepatitis C in Special Patient Cohorts: New Opportunities in Decompensated Liver Cirrhosis, End-Stage Renal Disease and Transplant Medicine

**DOI:** 10.3390/ijms160818033

**Published:** 2015-08-05

**Authors:** Anna Hüsing, Iyad Kabar, Hartmut H. Schmidt, Hauke S. Heinzow

**Affiliations:** Department of Transplant Medicine, University Hospital Muenster, 48149 Münster, Germany; E-Mails: anna.huesing@ukmuenster.de (A.H.); iyad.kabar@ukmuenster.de (I.K.); hauke.heinzow@ukmuenster.de (H.S.H.)

**Keywords:** HCV infection, chronic kidney disease, renal transplantation, decompensated liver cirrhosis, liver transplantation, new therapy regimen, DAA treatment

## Abstract

Worldwide, hepatitis C virus (HCV) is a common infection. Due to new antiviral approaches and the approval of direct-acting antiviral agents (DAA), HCV therapy has become more comfortable. Nevertheless, there are special patient groups, in whom treatment of HCV is still challenging. Due to only few data available, tolerability and efficacy of DAAs in special patient cohorts still remain unclear. Such special patient cohorts comprise HCV in patients with decompensated liver disease (Child-Pugh Class B or C), patients with chronic kidney disease, and patients on waiting lists to renal/liver transplantation or those with HCV recurrence after liver transplantation. HCV infection in these patient cohorts has been shown to be associated with increased morbidity and mortality and may lead to reduced graft survival after transplantation. Successful eradication of HCV results in a better outcome concerning liver-related complications and in a better clinical outcome of these patients. In this review, we analyze available data and results from recently published literature and provide an overview of current recommendations of HCV-therapy regimen in these special patient cohorts.

## 1. Introduction

Hepatitis C virus (HCV) infections are common worldwide. It is estimated that about 3% of the world’s population is infected by HCV, which results in 130–150 million carriers worldwide. According to the World Health Organization (WHO), 350,000 to 500,000 deaths per year are attributed to liver diseases caused by HCV. A sustained virological response (SVR) of HCV improves survival rates of HCV infected patients and lowers complication rates of HCV related liver disease. At the “American Association for the Study of Liver Diseases” (AASLD) meeting in November 2014, Hill and co-authors presented five-year observational data of 34,563 patients with and without SVR who had been treated with antivirals. The authors revealed that elimination of HCV resulted in a decrease of five-year-mortality of 62%–84%. According to their study, the risk for development of hepatocellular cancer (HCC) was lowered by 68%–79%; the risk for liver transplantation was strongly reduced and was lowered by 90% [1].

The launch of interferon-free regimens for the treatment of hepatitis c revolutionizes management of patients suffering from chronic hepatitis C infection. Response rates of more than 90%, improved tolerability and fewer side effects of the new drugs allow the treatment of patients who are contraindicated to interferon and who showed low response rates in interferon-based antiviral therapies. However, until now, few data are available exploring the tolerability and efficacy of direct-acting antiviral agents (DAA) in special patient cohorts. These include patients with decompensated liver disease (Child-Pugh Class B or C) or patients with HCV recurrence after liver transplantation. Moreover, advanced liver disease is commonly associated with advanced renal disease. High prevalence of HCV infection in patients with chronic kidney disease has been associated with increased morbidity and mortality in these patients. Therefore, HCV screening of patients suffering from chronic kidney disease before starting hemodialysis or consideration for transplantation is recommended.

Until now, only few data are available concerning the efficacy and safety of the new DAAs in special patient cohorts. Therefore, new studies and so-called “real-life”-experience reports from hepatitis registries are required to clarify safety and efficacy in special patient cohorts such as kidney and/or liver transplant recipients, patients suffering from end-stage renal disease, or liver cirrhosis.

In this review, we will explore these special patient cohorts and examine the current state of knowledge in this field. To establish further understanding, a comprehensive summary of pharmacokinetics of all the new DAAs in the setting of hepatic and renal impairment is added (Table 1).

### 1.1. Data Sources

A literature search was performed by looking for the search terms hepatitis C, HCV, renal insufficiency, renal impairment, chronic kidney disease, liver cirrhosis, end stage liver disease and liver transplantation. Only English studies from 1995 through 2015 were considered. In addition, further references and studies presented at the American Association for the Study of Liver Disease (AASLD) and the European Association for the Study of the Liver (EASL) congress as well from trials registered in clinicaltrials.gov were considered.

**Table 1:** Pharmacokinetics of direct-acting antiviral agents in hepatitis C treatment.

Antiviral Agent	Mechanism	Dosage	Absorption	Cmax	Metabolism	Elimination	Hepatic Impairment	Renal Impairment	Protein Bound	t_½_ (h)	Enzymes Involed in Metabolism
Simeprevir	NS3/4A protease inhibitor	150 mg oral daily with food	Bioavailability 60% with food, take with food.	After 4–6 h	Hepatic; saturable first pass metabolism	Biliary excretion; 91% Renal < 1%	Use in decompensated liver cirrhosis or moderate to severe hepatic impairment is not recommended.	In patients with mild, moderate or severe renal impairment no dose adjustment is required. Data missing exploring the safety in end-stage renal disease and hemodialysis.	>99%	110–130 in healthy volunteers; 41 in HCV-infected patients	Simeprevir is metabolized via CYP2A4 enzymes. Therefore, comedication with strong inducers or inhibitors of this enzyme is not recommended. Simeprevir is a mild inhibitor of CYP1A2 and intestinal CYP3A4. No effect on CYP2C9 and 2C19 and 2D6 are documented.
Sofosbuvir	NS5B nucleotide HCV RNA polymerase inihibitor	400 mg oral daily	Bio-availability not determined. Take with or without food.	After 1 h	Hepatic prodrug hydrolyzed to active metabolite GS-461203; dephosphorylated to predominant metabolite GS-331007	Urine 80% (3.5% SOF, 78% GS-331007) feces, 14%	Hepatic impairment and liver cirrhosis do not have an effect on the AUC of Sofosbuvir and its metabolite.	No dose adjustments are required for mild or moderate renal impairment (CrCl > 30 mL/min) (but safety and efficacy have not been established in patients with severe renal impairment (CrCl < 30 mL/min) or ESRD requiring hemodialysis	61%–65%	Parent drug: 0.4–0.75 major circulating metabolite, GS-331007: 27	The metabolism is CYP450 enzyme independent.
Ledipasvir	NS5A inhibitor	90 mg oral daily	Bio-availability 32%–53%; solubility is pH-depentend, take with or without food.	After 4–6 h	Hepatic, minimal; not CYP450 mediated	Feces 70%; Urine < 1%	No dose adjustment is required in hepatic impaired patients.	In patients with renal impairment no dose adjustment is required. but safety and efficacy have not been established in patients with ESRD requiring hemodialysis.	>99.8%	50	The metabolism is CYP450 enzyme independent.
Daclatasvir	NS5A replication Complex inhibitor	60 mg oral daily	Bio-availability not determined. Take with or without food.	After 1–2 h	Hepatic	Feces, 88%; Urine, 7%	No dose adjustment is Required in hepatic impaired patients.	No dose adjustment is required in renal impaired patients.	95.6%	12–15	Daclatasvir is substrate for CYP3A4 and P-gp. Therefore, comedication including CYP3A4 and P-gp inducers is contraindicated. Daclatasvir is a moderate inhibitor of P-gp, BCRP and OATP1B1/3 and shows limited inhibitory effects on CYP3A4.
(Paritaprevir/Ritonavir/Ombitasvir/Dasabuvir)	NS3/4A HCV protease inhibitor/ HIV protease inhibitor/ NS5A inhibitor + non-nucleoside HCV polymerase inihibitor	2 tablets of 75 mg/50 mg/12.5 mg oral daily + 250 mg twice daily	Bio-Availability, 70% for Dasabuvir, not determeined for other agents, take with food.	After 4–5 h	Hepatic	Feces > 86%	In mild hepatic impairment (Child Pugh A) no dose adjustment is required. In moderate impairment (Child Pugh B) it is not recommended, in Child Pugh C it is contraindicated.	No dose adjustment is required in renal impaired patients. The regimen has not been adequately studied in ESRD and hemodialysis patients.	Dasabuvir > 99.5%, Ombitasvir 99.9%, Paritaprevir 97%–98%, RTV > 99%	5.5/4/23 + 6	Paritaprevir inhibits OAT1Ba transporters and is metabolized via CYP3A4. Ritonavir inhibits CYP3A4. In combination it is used as a booster for concentrations of Paritaprevir. Ritonavir is a substrate, inhibitor and inducer of many enzymes and proteins. Dasabuvir is metabolized via CYP2C8 and CYP3A4. Ombitasvir is metabolized via hydrolysis and oxidation reactions.

Drug information based on summary of product information and according to Burgess S *et al.* [2].

### 1.2. Pharmacokinetics of Newly Available DAAs

Table 1 briefly summarizes the pharmacokinetics of the new DAAS and provides clinicians with a brief understanding for potential pittfalls of DAA use in special patient cohorts of end-stage renal disease, decompensated liver cirrhosis and transplant medicine. For a more detailed overview of pharmacokinetics we refer to Burgess *et al* [2].

### 1.3. HCV Infection in Patients with Chronic Kidney Disease Stage 4 or 5 Including Candidates for Renal Transplantation

Based on epidemiological studies, chronic HCV infection is known as an independent risk factor for the development of chronic kidney injury compared with patients without HCV [3,4]. Chronic HCV infection is associated with histological–pathological lesions in both native and transplanted kidneys. The relationship between positive HCV serology and chronic kidney injury is discussed, but considered controversial, in the literature. Immunological and non-immunological mechanisms leading to chronic kidney injury in HCV infected patients are discussed in the literature. The most common HCV-associated nephropathy is type 1 membrano-proliferative glomerulonephritis. Another manifestation is type II mixed cryoglobulinemia, which is related to HCV-containing immune complex deposition in the glomeruli. Other studies postulate that infection with HCV *per se* is associated with an increased risk for the development of renal insufficiency or proteinuria. Some connect proteinuria to be caused by a metabolic syndrome, which shows a higher prevalence in HCV-infected patients than in the general population. Another hypothesis postulates that chronic renal insufficiency in HCV infected patients could be the result of accelerated atherosclerosis promoted by HCV [5,6]. However, the mechanisms leading to chronic kidney disease in chronically HCV-infected patients are still unclear. Nevertheless, chronic HCV infection worldwide occurs in 20%–25% of patients suffering from chronic kidney disease. Furthermore, HCV infection is also associated with an increased morbidity and mortality in kidney transplant recipients [7,8,9]. According to several studies, 10%–25% of candidates considered for kidney transplantation concurrently suffer from advanced liver fibrosis or cirrhosis [10,11]. Moreover, kidney transplanted patients with HCV infection show higher rates of liver related complications and lower survival rates after transplantation than HCV-negative patients [7,12,13,14]. According to literature, 8%–28% of kidney transplant recipients die due to chronic liver diseases [15]. In patients with chronic kidney injury, specific treatment of HCV prior to kidney transplantation results in a better graft function and improved survival rates after transplantation [12]. According to a risk analysis, an active HCV-replication at the time of transplantation was identified to be an independent risk factor for a subsequent kidney graft failure [16]. Consequently, HCV treatment before kidney transplantation may avoid abovementioned complications, thus Patients considered for kidney transplantation should be prioritized for treatment.

Historically, PEG-interferon based treatment regimen was associated with low virological response rates in patients with end-stage kidney disease. Moreover, antiviral treatment including PEG-interferon after kidney transplantation is relatively contraindicated due to higher graft rejection rates [17,18]. Approval of new DAAs is highly promising for effective future treatment in these patients. However, until now, there are only few data exploring new antiviral treatment options in chronically kidney injured patients (GFR < 30 mL/min or hemodialysis) and in kidney transplant recipients. The 3D combination (Paritaprevir/Ritonavir/Ombitasvir and Dasabuvir) approved in January 2015 has been reviewed in phase II studies according to their pharmacokinetics and safety profile in patients with low-grade, moderate, and severe kidney injury. Results showed no clinical relevant modifications [19]. Preliminary results of an ongoing phase III study (ClinicalTrials.gov Identifier: NCT02207088) presented at the European Liver Congress (EASL) 2015 [20] seem to confirm the efficacy and safety of the 3D combination (Table 2 and Table 3). So far, 20 non-cirrhotic patients are currently included, 13 of them on dialysis. Patients are treated for 12 weeks with the 3D regimen Paritaprevir/Ritonavir/Ombitasvir plus Dasabuvir. Patients with GT1a additionally receive 200 mg of ribavirin each, four hours before dialysis. All 20 patients are currently HCV-PCR negative and 10 patients have achieved sustained virological response (SVR) rates at Week 4 after stopping treatment. Furthermore, the compatibility of the medication is good. In eight patients, ribavirin had to be interrupted. Four of the patients were given erythropoetin in the further course. Only in one patient the hemoglobin level dropped below <8 g/dL. Preliminary pharmacokinetic data show mean *C*_trough_ values of DAAs with stage 4 and 5 chronic kidney disease comparable to those without renal impairment, suggesting a good safety profile.

No final data are available investigating the treatment with the NSB5-polymerase-inhibitor Sofosbuvir (SOF) in patients with severe kidney injury or hemodialysis. Currently, there is an ongoing clinical phase IIb study (ClinicalTrials.gov Identifier: NCT01958281) examining the safety and effectiveness of the combined use of Sofosbuvir + Ribavirin (RBV) in HCV genotypes 1 and 3 patients with end stage kidney injury (Table 3). First results show comparable Sofosbuvir levels and four-times higher plasma levels of its metabolite GS-331007 in comparison to HCV patients without kidney injury. No therapy limiting severe side effects were observed [21]. However, the lower dose of Sofosbuvir (200 mg per day) and ribavirin (200 mg per day) in a 24-week course might result in low SVR rates of 40% (4 patients out of 10) in preliminary analysis.

Furthermore, not yet released Grazoprevir in combination with Elbasvir has been evaluated in a phase-3 study [22] with HCV genotype 1 infected patients (Table 3). Seventy-six percent of patients were on hemodialysis and 19% had an eGFR of 15–29 mL/min. SVR12 rates of 116 patients are available. Patients received 12 weeks Grazoprevir and Elbasvir. Six out of 122 patients were withdrawn from the study for various non-therapy-associated reasons. Only one patient out of 116 patients that were treated for 12-weeks, suffered a relapse (SVR12 99%) (Table 2). Furthermore, the therapy was well tolerated with a reporting of serious adverse events in 14% of cases, thus holding potential for a further treatment option in HCV genotype 1 infected patients with chronic kidney disease.

**Table 2:** SVR4 and SVR12 rates of HCV treatment in CKD stages 4/5 according to Saxena Varun *et al.* and Roth David *et al.* [22,23]. * Preliminary data.

Study	HCV Genotype	Treatment	Patients ( *n*)	SVR4	SVR12
RUBY-I *	GT 1b and 1a	Paritaprevir/ritonavir + Ombitasvir + Dasabuvir (plus Ribavirin in GT 1a) for 12 weeks	10	100% (10/10)	100% (2/2)
C-Surfer	GT 1	Grazoprevir + Elbasvir for 12 weeks	116		99% (15/116)

However, outcomes of clinically conducted phase-II or -III studies often fail to match those observed in heterogenous “real-life” populations. Therefore, results of longitudinal observational HCV-studies or registries are very important to bridge the knowledge gap between investigation and “real-life” application. In comparison, “real-life” data from the longitudinal cohort study TARGET [23], reporting on 19 patients with severe kidney disease (stages 4/5) receiving either the combination of Sofosbuvir and Simeprevir (SMV) or the combination of Sofosbuvir plus Ribavirin or Sofosbuvir/Pegylated-Interferon and RBV, suggest high cure rates of 85% at the cost of more side effects, such as anemia, renal function deterioration and severe adverse events.

**Table 3:** Inclusion and exclusion criteria of revised clinical studies in patients with advanced renal impairment.

Study	Inclusion Criteria	Exclusion Criteria	HCV GT	Patients (*n*)	Therapy
RUBY-1	- Treatment-naive adults - Chronic kidney disease with eGFR <30 mL/min/1.73 m^2^	- Clinically significant comorbidity	GT 1	20 #	3D regimen for 12 weeks (+RBV in GT 1a)
	- HBV and HIV negative - Non-cirrhotic *	- Hemoglobin < 10 g/dL			
NCT01958281	- HBV or HIV negative - Cirrhosis determination at screening	- Prior null response to PEG + RBV	GT 1 or 3	10 #	SOF + RBV for 24 weeks
	- Treatment-naive and experienced adults - Chronic kidney disease with eGFR <30 mL/min/1.73 m^2^	- Current or prior history of hepatic decompensation			
	- Not on hemodialysis	- Clinically significant comorbidity			
C-Surfer	- Treatment-naive or experienced adults - Chronic kidney disease with eGFR <30 mL/min/1.73 m^2^, including patients on hemodialysis	- Current or prior history of hepatic decompensation	GT 1	235	Grazoprevir + Elbasvir for 12 weeks
	- Compensated cirrhosis allowed § - HBV and HIV negative	- Advanced liver cirrhosis			

Study oversight of revised clinical studies focusing on patients with advanced renal impairment. (* Histologic diagnosis (Metavir score ≤ 3; Ishak score ≤ 4), or Screening FibroScan < 14.6 kPa or APRI ≤ 2 or Fibro Test ≤ 0.72; # preliminary data; § Histologic diagnosis or Screening FibroScan or Fibro Test).

## 2. HCV Infection in Patients after Renal Transplantation with GFR >30 mL/min

According to actual guidelines, kidney transplant recipients with renal function GFR >30 mL/min can be treated similar to patients with chronic HCV without any kidney injury. However, in patients after organ transplantation drug interactions with immunosuppressive agents have to be taken into account.

Sofosbuvir, Daclatasvir (DAC) and Ledipasvir (LDV) show no or only minor effects on hepatic CYP3A4 enzymes [31,32]. Therefore, dose adjustment of common immunosuppressive agents like cyclosporine and tacrolimus is usually not required. However, serum levels of immunosuppressive agents need to be closely monitored during antiviral treatment. In case of combined use of Ritonavir-bossted protease inhibitor Paritaprevir, dose adjustment of tacrolimus and cyclosporine is required. Due to prolonged blood serum level duration of tacrolimus (7-fold higher) and cyclosporine (3-fold higher) under 3D-therapy regimen, dose adjustment is required depending on tacrolimus or ciclosporin trough levels.

## 3. Patients with Decompensated Liver Cirrhosis

The main target of anti-viral therapy in patients on the waiting list for liver transplantation is to prevent a HCV recurrence and reinfection of the graft [33]. Moreover, the number of organ donations remains critical in most transplant regions. Cure of hepatitis C may even result into withdrawal of selected patients on the transplant waiting list due to significant improved liver function. Depending on genotype, the historical therapy regimen combining Ribavirin und PEG-interferon showed only low response rates of about 20% in patients with end-stage liver cirrhosis [33,34,35]. Additionally, in many of these patients, treatment had to be interrupted due to therapy-related side effects such as cytopenia, infections and decompensation of cirrhosis. Interferon based therapy was highly associated with temporarily decreased liver function, which resulted in liver failure once therapy was administered at advanced stages of liver cirrhosis, resulting in only few studies exploring PEG-interferon-based therapy regimen in patients with Child Pugh Score >8. Overall PEG-interferon-based treatment cannot be recommended in this patient cohort [36].

However, with the approval of the latest generation DAAs like Sofosbuvir, Simeprevir, Daclatasvir, Ledipasvir and the 3D combination Paritaprevir/Ritonavir, Ombitasvir + Dasabuvir potent drugs for HCV therapy are now available. Clinical studies report response rates of these substances of >90% [37]. Nevertheless, there are only few data available exploring HCV treatment in patients with decompensated cirrhosis (Child-Pugh Class (CPC) A with evidence of portal hypertension or Child-Pugh class B or C). In the following, we summarize recent knowledge and treatment recommendations for this patient cohort.

Fourty-eight-week therapy using Ribavirin and Sofosbuvir in 50 cirrhotic patients with either portal hypertension (CPC A) or with decompensated cirrhosis (CPC B) was recently presented [24] (Table 4). HCV GT 1–4 were included. The overall SVR12 rate was 72% (33/46). SVR12 was 78% in CPC A and 68% in CPC B. Interestingly, the study also observed improvements of serum albumin, the MELD score and a decrease of bilirubin. Furthermore, a decrease of ≥10% in the hepatic venous pressure gradient (HVPG) was observed in 38% of patients, a decrease in HVPG of ≥20% in 24% of patients. Treatment was generally well tolerated and resulted in only a few cases to premature discontinuation of therapy due to adverse events (*n* = 4).

The effect of Sofosbuvir/Ledipasvir + Ribavirin and the combination of Sofosbuvir + Daclatasvir + Ribavirin have been investigated in the SOLAR-2 study [25] and the ALLY-1 study [26] (Table 4). Both studies included patients with advanced or decompensated cirrhosis. In the SOLAR-2 study [25] patients with HCV genotype 1 or 4 and CPC B or C were treated for 12 or 24 weeks. Preliminary results revealed high SVR rates of 85%–88%, irrespective of treatment duration in genotype 1, whereas longer treatment duration was superior (SVR rate of 86% *vs.* 57%) in genotype 4. The SOLAR-2 study could also show improvement of MELD- and Child-Pugh Score in patients treated with LDV/SOF + RBV suffering from decompensated cirrhosis and post-liver transplantation. However, the reporting of seven deaths, even though not considered as treatment related by the authors, raise the question of safety issues of DAAs in this special patient cohort since it may be difficult to differentiate between the natural course of disease or direct toxicity from DAA therapy being causative for death.

**Table 4:** Inclusion and exclusion criteria of revised clinical studies in patients with decompensated liver cirrhosis or post liver transplantation.

Study	Inclusion Criteria	Exclusion Criteria	HCV GT	Patients (*n*)	Therapy	Saftey (%)
NCT01687257 [24]	- Cirrhosis with Child-Pugh score < 10 - Esophageal or gastric varices on endoscopy - HVPG > 6 mmHg - BMI ≥ 18 kg/m^2^ - Treatment-naive to all nucleotides/nucleoside treatments for chronic HCV infection - eGFR > 50 mL/min/1.73 m - No history of hepatorenal or hepatopulmonal syndrome	- HIV or HBV co-infection - AFP > 50 unless negative imaging for hepatic masses within the last 6 months or during screening - Refractory ascites as defined by requiring paracentesis > twice within 1 month prior to screening - Active variceal bleeding within 6 months of screening	GT 1–4	50	SOF + RBV for 48 weeks	- AEs: 94% - Grade 3–4 AEs: 20% - Serious AEs: 22% - Treatment D/C due to AE: 9% - Death: 0%
SOLAR-2 [25]	- Treatment-naive or experienced adults - No hepatocellular carcinoma (HCC) - eGFR > 40 mL/min/1.73 m - eGFR > 40 mL/min/1.73 m - Platelets > 30,000/mL - CPC B or C cirrhosis - Or post LTx recurrence	- HBV or HIV co-infection - Previous treatment with DAA	GT 1 or 4	329	SOF + LDV + RBV for 12 or 24 weeks	- AEs: 94% - Grade 3–4 AEs: 25% - Serious AEs: 28% - Treatment D/C due to AE: 3% - Death: 4%
ALLY-1 [26]	- Treatment-naive or experienced adults - Advanced cirrhosis * - Post-transplant subjects at least 3 months post-transplant with no evidence of moderate or severe rejection	- HIV or HBV co-infection as documented by HBV - Active hospitalization for decompensated liver disease - Previous treatment with DAA	All GTs	113	SOF + DCV + RBV for 12 weeks	- AEs: 94% - Grade 3–4 AEs: 15% - Serious AEs: 13% - Treatment D/C due to AE: 2% - Death: 0%
IMPACT [27]	- Treatment-naive or experienced adults - CPC A cirrhosis # with evidence of portal hypertension (esophageal varices or HPVG ≥ 10 mmHg - CPC B cirrhosis #	- Previous treatment with DAA - HBV or HIV co-infection	GT 1 or 4	28	SOF + DCV + SMV	- AEs: 67% - Grade 3–4 AEs: 0% - Serious AEs: 0% - Treatment D/C due to AE: 0% - Death: 0%
CORAL-I [28]	- Post liver transplant - Treatment-naive or experienced adults either pre or post liver or renal transplant - Currently taking an immunosuppressant regimen based on either tacrolimus or cyclosporine. Corticosteroids such as prednisone or prednisolone are permitted as components of the immunosuppressant regimen providing the dose is not more than 10 mg/day	- HBV or HIV co-infection - Use of everolimus or sirolimus as part of immunosuppressive regimen - No severe fibrosis or cirrhosis (Metavir score ≤ F2)	GT 1	34	3D-regimen + RBV for 24 weeks	- AEs: 97% - Grade 3–4 AEs: 0% - Serious AEs: 6% - Treatment D/C due to AE: 3% - Death: 0%
NCT01938430 [29]	- Post liver transplant - Metavir F0–F3 - CPC A cirrhosis * - Total bilirubin ≤ 10 mg/dL, Hemoglobin ≥ 10 g/dL - eGFR > 40 mL/min/1.73 m - Platelets > 30,000/mL	- HBV or HIV co-infection	GT 1 or 4	223	SOF/LDV + RBV for 12 or 24 weeks	- AEs: 98% - Serious AEs: 20% - Treatment D/C due to AE: 3% - Death: 2%
		- Previous treatment with DAA				
SATURN [30]	- Post liver transplant - Treatment-naive or experienced adults - Stable immunosuppressant regimen with either tacrolimus or ciclosporin - Metavir-Score F1–F4	- HBV or HIV co-infection - Evidence of acute or chronic hepatic decompensation after LTx	GT 1b	35	SMV + DCV + RBV for 24 weeks	- AEs: 94% - Grade 3–4 AEs: 26% - Serious AEs: 20% - Treatment D/C due to AE: 6% - Death: 0%

Study oversight of revised clinical studies focusing on patients with decompensated liver cirrhosis or pos liver transplantation. (* FibroScan with kPa >12 or FibroTest score of >0.75 and APRI > 2 or liver biopsy documenting cirrhosis; # FibroScan with kPa >14.5).

The ALLY-1 phase-III study [26] (Table 4) included patients with cirrhosis (*n* = 60) and HCV GT 1-4. Patients were treated with the combination of Daclatasvir, Sofosbuvir and Ribavirin. Depending on Child-Pugh Class A-C SVR12 rates of 92%, 94% and 56% were achieved, respectively. Overall SVR12 rates by HCV GT irrespective of CPC showed best results in GT 1b of 100%, 76% in GT 1a; 80% in GT 2, 83% in GT 3 and 100% in GT 4, respectively.

The findings demonstrate that this combination yields high SVR rates in cirrhotic patients irrespective of the GT but that further studies are required to define the best therapy management for Child-Pugh C patients. Moreover, a serum albumin level <2.8 g/dL was also associated with a poor SVR12 rate of 56%, thus serving as a negative predictor. The treatment was overall well tolerated as summarized in Table 4.

**Figure 1:**
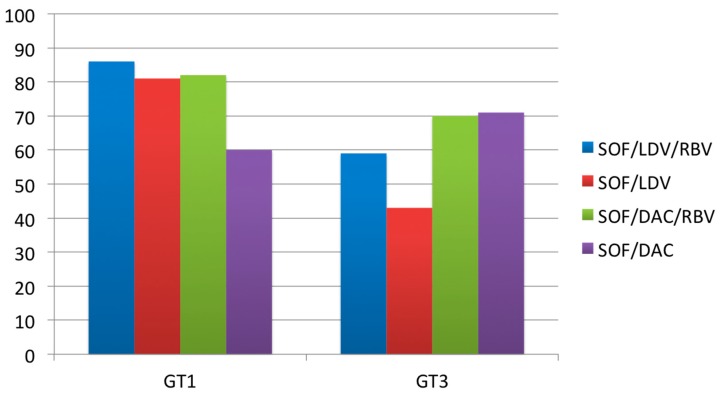
Adapted from Foster GR *et al.* [38]: Intention to treat analysis with SVR12 by genotype and therapy regime.

A related real-world National Health Service England (NHS E) funded observational study [38] investigated the efficacy of SOF combined with either LDV or DAC in mainly HCV GT 1 (235 patients) or GT 3 (189 patients) infected patients with decompensated liver cirrhosis (Child-Pugh Score > 7). Patients were treatment-naïve or experienced (Peg-IFN + RBV). The SVR12 rates by genotype and therapy regimen are shown in Figure 1. SOF/LDV with or without RBV, and SOF/DAC with RBV, did not work as well against GT 3 as they did against GT 1. However, SOF/DAC worked better for GT 3 and addition of RBV had no beneficial effect. Moreover, over 40% of patients showed improvement in liver function by means of improvement of >2 MELD score points. The rate of serious adverse events of 26% was similar in comparison to abovementioned clinical trials.

Interim analysis of a French multicenter compassionate use program of Daclatasvir plus Sofosbuvir with or without ribavirin in liver cirrhosis and HCV GT 3 report SVR4 rates of 76% (22/29 patients) in a 12 weeks therapy regimen *vs.* 88% (52/54 patients) in a 24 weeks regimens [39]. Unfortunately, subgroup analyses of Child-Pugh status in this patient cohort are not available yet, limiting treatment recommendation.

The ongoing IMPACT study (Phase-II) [27] (Table 4) analyses a 12-week treatment of the combination of Sofosbuvir, Daclatasvir and Simeprevir in patients with HCV GT 1 or 4 and decompensated liver cirrhosis. So far, 28 patients with CPC B or CPC A with evidence of portal hypertension have been evaluated. All patients with available data have achieved SVR4 (CPC A 12/12; CPC B 2/2). Tolerability of the treatment was good. Moreover, pharmacokinetic analysis revealed SMV exposures in CPC B patients to be within the range observed for CPC A patients, thus, in contrast to the label of SMV, suggesting possible use in decompensated cirrhosis.

As for the 3D regimen (Paritaprevir/Ritonavir + Ombitasvir + Dasabuvir ± Ribavirin), no data on treatment in HCV GT 1 with decompensated cirrhosis are available yet. However, an integrated analysis of six phase-III trials of GT 1 patients receiving Ombitasvir/Paritaprevir/r, Dasabuvir with or without Ribavirin (RBV) for 12 and 24 weeks have shown excellent SVR12 rates in compensated cirrhosis (Child-Pugh Score < 6) of 96% (*n* = 189 patients) [40]. As results from a *post-hoc* analysis of TURQUOISE-II [41], which did not include cirrhotic patients, implicate an improvement in total bilirubin levels, α-fetoprotein levels (AFP), international normalized ratio (INR), absolute platelet count and serum albumin levels after a 12 to 24 week treatment with the 3D regimen, an improvement in liver function might also be assumed for the special patient cohort of patients with advanced cirrhosis. To what extent these results are applicable to patients with advanced cirrhosis is currently evaluated in the TURQUOISE-CPB study (ClinicalTrials.gov Identifier: NCT02219477).

However, outcomes of clinically conducted phase-II or -III studies often fail to match those observed in heterogenous “real-life” populations. Therefore, results of longitudinal observational HCV-studies or registries are very important to bridge the knowledge gap between investigation and “real-life” application.

**Figure 2:**
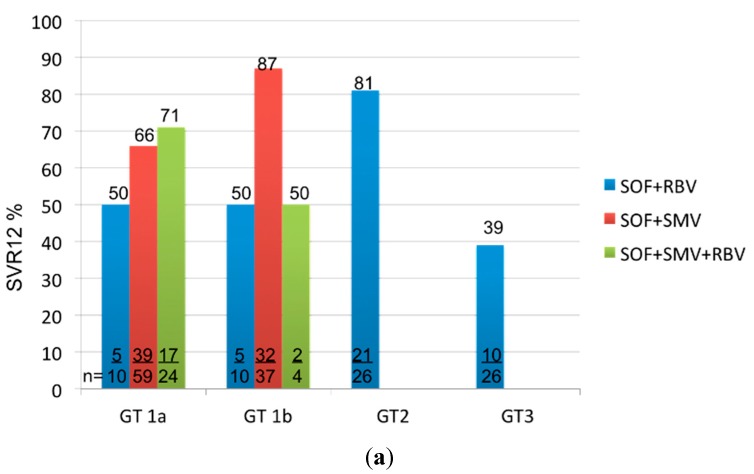

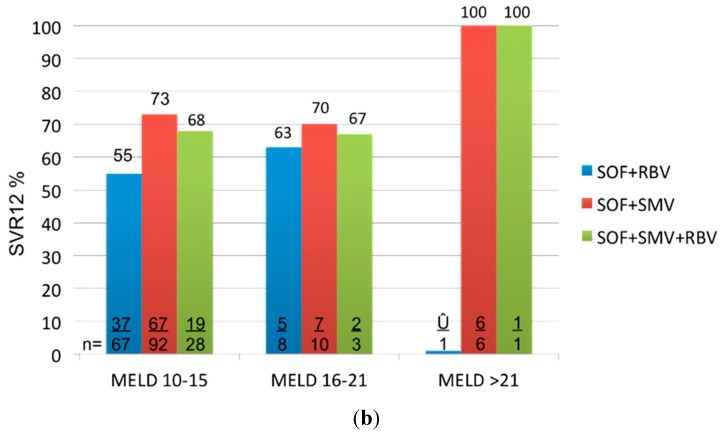
Adapted from Reddy *et al.* [42]: SVR12 by Genotype (**a**); and MELD Score (**b**) depending on treatment regimen.

Interim results from the longitudinal, observational HCV-TARGET study [42] have included a total of 256 patients (*n* = 183 HCV GT1; *n* = 30 HCV GT2; *n* = 33 HCV GT3) with liver cirrhosis (*n* = 219 Model of Endstage Liver Disease (MELD) Score 10–15; *n* = 29 MELD Score 16–21; *n* = 10 MELD Score > 21) so far. SVR12 rates of 216 patients are available at date. Patients have either received a combination of SOF + RBV (*n* = 76), SOF + SMV (*n* = 108) or the combination of SOF + SMV plus RBV (*n* = 32). Overall SVR12 rates depending on the genotype or MELD Score are demonstrated in Figure 2.

Moreover, the authors documented an improvement of bilirubin and albumin levels in 80% and 61% of patients, respectively. Twenty-six of their patients had baseline MELD and post treatment data available. Of them, 18 had improvement, five unchanged, and three worsened after four weeks of treatment. The authors showed that markers of hepatic and synthetic function improved during the short-term follow-up.

Furthermore, final evaluation of 954 “real-life” HCV patients treated with 12 weeks regimens containing SOF ± SMV in the TRIO Health program have recently been presented at EASL 2015 [43]. 290 patients with cirrhosis were included, receiving either 12 weeks of Peg-Interferon + RBV + SOF, RBV + SOF or SMV + SOF ± RBV. Table 5 overviews the SVR12 rates. Subgroup analysis of cirrhosis stage and its correlation with SVR rates unfortunately have not been published yet.

**Table 5:** Per protocol analysis of SVR12 rates in the TRIO Health Program.

Genotype	PEG + RBV + SOF	SMV + SOF ± RBV	SOF + RBV
GT1	77% (*n* = 54/70)	83% (*n* = 110/133)	n.a.
GT2	n.a.	n.a.	93% (*n* = 140/150)

Adapted from Dieterich *et al.* [43]: Per protocol analysis of SVR12 rates in HCV GT1 or GT2 infected patients with liver cirrhosis treated with 12 weeks regimens containing Sofosbuvir ± Simeprevir in the TRIO Health program.

As for now, according to the results of the abovementioned studies and currently available EASL and AASLD recommendations but also in awareness of yet limited available data we suggest following therapy regimens in patients with decompensated liver cirrhosis: (Table 6).

**Table 6:** Treatment recommendations for patients with decompensated liver cirrhosis.

HCV Genotype	Therapy
**GT 1**	● Ledipasvir + Sofosbuvir + Ribavirin for 12 weeks. In case of Ribavirin intolerance treatment prolongation up to 24 weeks should be evaluated. ● Simeprevir + Sofosbuvir for 12 weeks.
**GT 2**	● Sofosbuvir + Ribavirin for up to 48 weeks.
**GT 3**	● Sofosbuvir + Daclatasvir + Ribavirin for 24 weeks.
**GT 4**	● Ledipasvir + Sofosbuvir + Ribavirin for 24 weeks.
**GT 5/6**	● No data available.

Recommendations for patients with decompensated liver cirrhosis. Since drug approval of the DAA’s vary, health care cost recovery should be evaluated prior to treatment start. Recommendations are given for DAA combinations with most data available at the moment.

## 4. Patients with HCV-Recurrence after Liver Transplantation

HCV related liver cirrhosis with or without HCC is the foremost indication for liver transplantation within the UNOS regions and Eurotransplant [44]. HCV recurrence in patients with detectable HCV-RNA at the time of transplantation is somewhat of 100%. In patients who do not receive antiviral treatment, liver cirrhosis occurs in up to 25% within five years of liver transplantation and long-term survival is reduced by 25% compared to patients with other diagnosis leading to liver transplantation [45,46]. Therefore, HCV infection in liver transplant recipients should be close monitored and treated. Up to now, the standard therapy regimen combining Pegylated-Interferon and Ribavirin showed marginal response rates of 30%–40% [47,48,49] in this patient cohort. Furthermore, interferon-containing regimens also contain a higher risk of toxic effects in liver transplant recipients who receive immunosuppressive therapy that might lead to termination of antiviral therapy. Approval of new potent DAAs provides hope that sustainable HCV healing in liver transplant recipients may become feasible. Preliminary data from TARGET-cohort analyzing HCV treatment in liver transplant recipients with genotype 1 show SVR in 90% of patients who received Sofosbuvir/Simeprevir with or without Ribavirin 4 weeks after finishing treatment (SVR4) [50]. The ongoing CORAL-1 (Table 4) study exploring HCV non-cirrhotic genotype 1 liver transplant recipients treated with 3D-combination (Paritaprevir/Ritonavir, Ombitasvir and Dasabuvir) + Ribavirin for 24 weeks demonstrated SVR rates of 97% [28]. First data (NCT01938430) available for liver-transplant recipients receiving a combination of Sofosbuvir/Ledipasvir + Ribavirin (Table 4) also revealed excellent results. Therapy was well tolerated, even in patients with decompensated cirrhosis. Deaths occurred in 2% of cases but were not considered as therapy related. In patients with low-grade liver fibrosis (grade F0–F3) as well as in patients with compensated liver cirrhosis (Child-Pugh Class A), 12- and 24-weeks of therapy, respectively, led to response rates of 96%, whereas patients with Child B or C liver cirrhosis showed lower SVR rates of 85% and 65%, respectively [29]. The authors also documented that SVR12 in patients with decompensated cirrhosis is associated with improvements in CPC and MELD scores, indicating that HCV clearance can improve hepatic function by diminishing HCV-related liver inflammation.

The ALLY-1 phase-III study [26] also included patients with HCV recurrence after liver transplantation (*n* = 53). Thirty precent of the patients also had a Metavir Score F4 at the time of inclusion. Patients with HCV GT 1–6 (77% GT1, 21% GT3, 2% GT6) were treated with Daclatasvir + Sofosbuvir + Ribavirin for 12 weeks. Ninety-four percent of liver transplant recipients with HCV recurrence achieved SVR12. Moreover, due to a favorable drug-drug interaction profile no dose modification of immunosuppressant medication was necessary in this patient cohort, suggesting a high efficacy and safety of this treatment combination for this special patient cohort.

Preliminary results of 35 patients in the on-going phase-II study SATURN [30] (Table 4), investigating the combination of SMV + DAC + RBV in patients with recurrent HCV GT 1b infection after liver transplantation, including non- and cirrhotic patients, are showing high SVR4 rates of 90%–93%, also demonstrating promising results in this patient cohort. Simeprevir should not be administered with cyclosporine. Safety and tolerability were reported to be good with only few grade 3–4 adverse events.

Based on the currently available data, DAAs seem to be safe and effective in the treatment of liver transplant recipients.

According to available data, we suggest starting Ledipasvir/Sofosbuvir ± Ribavirin therapy early in liver transplant patients with recurrence of HCV genotype 1 and 4 (Table 7). To date, there are no data available concerning the combined use of sirolimus and everolimus inhibitors and Ledipasvir. Additionally, no data for the interaction-potential for mycophenolate mofetil, mycophenolic acid, or azathioprine and Ledipasvir exist but it is expected that Ledipasvir and Sofosbuvir do not have interactions with these immunosuppressive agents.

**Table 7:** Treatment recommendations for HCV recurrence after liver transplantation.

HCV Genotype	Therapy
**GT 1**	● Ledipasvir + Sofosbuvir + Ribavirin for 12 weeks. In case of Ribavirin intolerance treatment prolongation up to 24 weeks should be evaluated. ● Simeprevir + Sofosbuvir ± Ribavirin for 12 weeks. ● Paritaprevir, ritonavir, Ombitasvir and Dasabuvir with Ribavirin for 12 weeks (genotype 1b) or 24 weeks (genotype 1a with cirrhosis).
**GT 2**	● Sofosbuvir + Ribavirin for 12 weeks (also accounts for patients with post-transplant compensated cirrhosis). In decompensated cirrhosis prolongation up to 24 weeks should be evaluated.
**GT 3**	● Sofosbuvir + Daclatasvir + Ribavirin for 12–24 weeks depending on presence of post-transplant cirrhosis.
**GT 4**	● Ledipasvir + Sofosbuvir + Ribavirin for 24 weeks. ● Paritaprevir, ritonavir and Ombitasvir plus Ribavirin for 12 or 24 weeks depending on presence of post-transplant cirrhosis. ● Sofosbuvir + Simeprevir ± RBV for 12 weeks.
**GT 5/6**	● No data available.

Recommendations for patients with HCV recurrence after liver transplantation. Since drug approval of the DAAs vary, health care cost recovery should be evaluated prior to treatment start. Recommendations are given for DAA combinations with most data available at the moment.

In genotype 1 and 4, the 3D regimen can also be administered leading to comparable response rates (Table 7). Special attention should be given to drug interactions caused by ritonavir. Therefore, levels of immunosuppressive agents (such as tacrolimus or cyclosporine) have to be monitored closely and dose adjustments should be considered during therapy with such a booster protease inhibitor. Based on the TARGET study [50] the combination of Simeprevir + Sofosbuvir ± Ribavirin leads to high SVR4 rates of 90% and thus can also be evaluated. In genotype 2 and 3, we suggest a primary regimen using the combination Sofosbuvir + Ribavirin for 24 weeks. In genotype 3 the combination of Sofosbuvir + Daclatasvir + Ribavirin for 12 weeks can be applied alternatively.

Table 7 gives and overview of treatment recommendations for this special patient cohort.

## 5. Conclusions

According to the results of studies available to date, the use of DAAs in patients with CKD stages 4/5, decompensated liver cirrhosis and in those with HCV recurrence after renal and liver transplantation seems reasonable.

In liver transplant recipients who show reinfection, we suggest starting antiviral therapy early in the course. We recommend interaction checks for the administered medication especially a close monitoring of immunosuppressive agents and measurement of their blood levels to prevent toxicity or rejections. Moreover, based on the excellent response rates of HCV under new therapy regimen one should discuss new opportunities of recruiting liver organs in times of organ shortage. In case of considering HCV-positive organs for extended donor criteria waiting times could be reduced, e.g., in patients who are not sufficiently represented by the MELD score. Thus, risk of dying during the waiting time period might be reduced in these patients.

Despite the lack of data concerning antiviral treatment in patients with chronic kidney disease, the EASL [37] has already taken up this issue suggesting antiviral treatment in chronic kidney disease, particularly those who are suitable candidates for renal transplantation. Until more data are available, antiviral treatment of HCV patients with GFR < 30 mL/min or hemodialysis should exclusively be performed in specialized centers. According to our experience, treatment is feasible in individual cases; however, plasma level monitoring may be a helpful tool to manage the treatment. Simeprevir, Daclatasvir and the 3D combination can be considered. Depending on cirrhosis status 12 or 24 week therapy regimens should be applied. Moreover, in case of Ribavirin administration, individualized dosing is recommended.

For possible interactions with co-medication, we recommend www.hep-druginteractions.org, which provides an overview about possible interactions of DAAs and is being timely updated after new drug approvals.
